# Supplementary material for: foxQ2 marks fast-acting interneurons including dopaminergic neurons of mushroom bodies and central complex in the beetle Tribolium castaneum
Source: eLife. 2026 Oct 27;15:RP111703. doi: 10.7554/eLife.111703 (PMC13713031; doi:10.7554/eLife.111703)
Supplement: Peer review history

# Editor report: eLife Assessment

**DOI:** 10.7554/eLife.111703.3.sa0

**Authors:** Gáspár Jékely

**Affiliations:** Heidelberg University Germany

**Keywords:** Convincing, Valuable

This **valuable** descriptive study describes the expression of a developmentally relevant transcription factor in the adult *Tribolium* brain. The evidence supporting the claims is **convincing** and based on a very detailed and rigorous analysis of light microscopy data, which, however, lacks single-cell resolution. This neuroanatomical study is of interest to the field of insect neural development and neuroscience.

# Referee report: Reviewer #1 (Public review)

**DOI:** 10.7554/eLife.111703.3.sa1

[Editors' note: The reviewing editor has assessed the revisions. The authors have addressed the previous minor concerns of the reviewers, added more details on the generation of the brainbow constructs and have made the image stacks available on public repositories.]

Summary:

Pang et al. investigated the expression pattern of the transcription factor foxQ2II in an adult beetle brain. They find nine distinct clusters, with many neurons expressing Glut/ChaT and dopamine. Some of the dopamine neurons resemble cell types described in Drosophila. Several neurons seem to project to prominent higher brain regions such as the MB and CX, and might even connect to both.

Strengths:

The authors use state-of-the-art labeling techniques for the analysis of individual cell types, such as beetle brainbow, to investigate the until now unknown expression of the transcription factor in the adult beetle brain.

Rigorous cell reconstruction and image analysis revealed a better understanding of the anatomy of the labeled cells.

# Referee report: Reviewer #2 (Public review)

**DOI:** 10.7554/eLife.111703.3.sa2

Summary:

The authors provide the first thorough profiling of neurons in *Tribolium* characterized by the expression of the transcription factor foxQ2, which will be useful for developmental neurobiology. They use state-of-the-art methods convincingly to not only identify the neurons, but also to further characterize them anatomically and neurochemically.

Strengths:

Thorough and meticulous application of state-of-the-art anatomical methods in a non-standard laboratory organism.

# Author comment: Author response

**DOI:** 10.7554/eLife.111703.3.sa3

**Authors:** Ying Pang, Bastian Klußmann-Fricke, Doga Cedden, Jiajun Zhang, Johannes B Schinko, Michalis Averof, Thomas Dieter Riemensperger, Gregor Bucher

**Affiliations:** University of Göttingen Göttingen Germany; abberior GmbH Göttingen Germany; University of Göttingen Göttingen Germany; University of Cologne Cologne Germany; University of Göttingen Göttingen Germany; Centre National pour la Recherche Scientifique et Technique (CNRST) Lyon France; University of Cologne Cologne Germany; University of Göttingen Göttingen Germany

The following is the authors’ response to the original reviews.

**Public Reviews:**

**Reviewer #1 (Public review):**

Summary:

Pang et al. investigated the expression pattern of the transcription factor foxQ2II in an adult beetle brain. They find nine distinct clusters, with many neurons expressing Glut/ChaT and dopamine. Some of the dopamine neurons resemble cell types described in Drosophila. Several neurons seem to project to prominent higher brain regions such as the MB and CX, and might even connect to both.

Strengths:

The authors use state-of-the-art labeling techniques for the analysis of individual cell types, such as beetle brainbow, to investigate the until now unknown expression of the transcription factor in the adult beetle brain.

We would want to add that this work establishes and introduces the brainbow system for the first time in an arthropod outside *Drosophila melanogaster* and that we are the first (outside flies) to relate the expression of a neural transcription factor with neural projection and neurotransmitter content.

Rigorous cell reconstruction and image analysis revealed a better understanding of the anatomy of the labeled cells.

Weaknesses:

The brainbow labeling seems to include all cells labeled by the enhancer trap line, as well as the ones not expressing foxQ2II. Thus, it is unclear how useful this data is to compare individual cells to other insects.

We kindly disagree with the first statement: not all cells of the enhancer trap are labelled but a subset. Therefore, we call it “sparse labelling” in our manuscript while we do not reach “single-cell labelling”, which admittedly limits precision.

The functional relevance of this transcription factor in the adult brain cell is still unknown. It is therefore unclear if the described neurons have any specific function and if they require this transcription factor for normal function.

Previously, we published that this gene has an important function in neural development during embryogenesis. Actually, we have done extensive RNAi experiments to test for an effect during postembryonic development. We found surprisingly small defects when looking at alterations in several imaging lines. However, we found some changes in behavior. Given the extensive data presented in the current paper, we decided to publish these functional data (another 12 figures/suppl. figures) separately.

We also note that the identity/function of neurons is determined by a mix of transcription factors. Disentangling the individual role of each of those transcription factors indeed is an exciting question but a major endeavor beyond the scope of this paper.

Overall, the neural reconstructions are missing single-neuron details; it is difficult to compare the shown cell types to specific cell types in Drosophila based on the presented data, and this finding remains speculative.

Indeed, we do not reach single-cell resolution, which is below the standards of fly neurobiology. However, compared with all other arthropods we reach a unique level of precision. Specifically, we are the only ones outside fly research that relate the expression of a developmental transcription factor to neural projection and neurotransmitter content.

We also think that combining our transgenic line with dopamine expression was sufficient to compare the labelled cells to fly neurons. From what we saw in that analysis, we feel that most homology assessments of single neurons across such large evolutionary distances will remain hypothetical to some degree.

**Reviewer #2 (Public review):**

Summary:

The authors provide the first thorough profiling of neurons in *Tribolium* characterized by the expression of the transcription factor foxQ2, which will be useful for developmental neurobiology. They use state-of-the-art methods convincingly to not only identify the neurons, but also to further characterize them anatomically and neurochemically.

Strengths:

Thorough and meticulous application of state-of-the-art anatomical methods in a nonstandard laboratory organism.

Thank you for this encouraging comment.

Weaknesses:

No weaknesses were identified by this reviewer.

Comments:

I don't really have any major suggestions at all. Loved the work. There is only one tiny nitpicking aspect:

P21: "Biogenic amines are involved in learning and memory and setting arousal threshholds (Davis, 2023), which are functions performed by the mushroom bodies and related to the function of the central complex in goal directed navigation, respectively." MBs mainly process olfactory memory. At least in Drosophila, most other kinds of memories are being supported elsewhere. https://pubmed.ncbi.nlm.nih.gov/10454381/ such as, e.g., visual pattern learning in the CX https://pubmed.ncbi.nlm.nih.gov/16452971/ or motor learning in motor neurons https://pubmed.ncbi.nlm.nih.gov/38779314/ or ventral ganglion, antennal lobes, and median bundle for place learning: https://pubmed.ncbi.nlm.nih.gov/10706599/

If the authors focus on MBs, this sentence ought to reflect the fact that the function of the MBs is much narrower than the current sentence appears to suggest.

Thanks for this clarification – we have rephrased:

"Biogenic amines are involved in learning and memory and setting arousal thresholds (Davis, 2023). This relates to the mushroom bodies’ function in olfactory memory, and the function of the central complex in visual pattern learning and goal-directed navigation, respectively."

**Recommendations for the authors:**

**Reviewer #1 (Recommendations for the authors):**

(1) The authors claim that several dop-positive cell types resemble cell types described in Drosophila - to be able to better compare the two, it would be great to have the supplement and main figure pictures in one figure.

We have added Fig. 11 from the main text to suppl. Fig. 3 for comparison

(2) In Figure 11 and the corresponding supplement, it would be great to have some landmarks to better understand the expression patterns.

In the legend, we have now referred to Figs. 3, 4 and 5 for depictions of these neurons within the neuropil reconstructions

(3) For a better understanding of neurotransmitter expression, is it possible to figure out if dopamine is rather coexpressed with Glut- or ChaT-positive neurons?

Very interesting idea. Unfortunately, the first author of the study has graduated and left the lab, such that we are unable to add this piece of information.
